# The Role of Femoral Head Size and Femoral Head Coverage in Dogs with and without Hip Dysplasia

**DOI:** 10.3390/vetsci10020120

**Published:** 2023-02-04

**Authors:** Mehmet Pilli, Deniz Seyrek Intas, Ilker Etikan, Pelin Yigitgor, Martin Kramer, Bernd Tellhelm, Kerstin von Puckler

**Affiliations:** 1 Department of Surgery, Faculty of Veterinary Medicine, Near East University, Near East Avenue, Nicosia 99010, Turkey; 2 Department of Biostatistics, Faculty of Medicine, Near East University, Near East Avenue, Nicosia 99010, Turkey; 3 Department of Surgery, Faculty of Veterinary Medicine, Bursa Uludag University, Gorukle Campus, Nilufer, Bursa 16059, Turkey; 4 Small Animal Clinic, Faculty of Veterinary Medicine, Justus-Liebig University, 35392 Giessen, Germany

**Keywords:** coverage, dog, femoral head area

## Abstract

**Simple Summary:**

Canine hip dysplasia is a nonhealing developmental orthopedic disorder resulting in osteoarthrosis of the hip joints and lameness. Radiography is an important tool to diagnose, grade and assess prognosis in hip dysplasia. The purpose of this study was to investigate radiographically detectable and measurable parameters that could indicate a predisposition to hip dysplasia. Radiographs of 264 dogs presented for canine hip dysplasia screening were evaluated for femoral head size, coverage of the femoral head by the acetabulum and acetabular length in relation to dysplasia status according to the Fédération Cynologique Internationale. No significant relationship between femoral head area and Fédération Cynologique Internationale assessment was detected. Femoral head area was breed-specific and larger in non-dysplastic dogs, males and German wirehaired pointers. Coverage of the femoral head by the acetabulum was significantly affected by presence of dysplasia and breed. All breeds and both sexes showed strong positive correlations between femoral head area and acetabular length.

**Abstract:**

The subject of hip dysplasia in dogs is still current and preoccupies both animal owners and veterinarians. Major factors affecting the development of the disorder are hip laxity and incongruent joints. Many studies on etiology, pathogenesis, and early diagnosis have been performed to reduce prevalence and select healthy dogs for breeding. The purpose of the present study was to investigate a possible relationship between dysplasia and femoral head area (FHA), femoral coverage by the acetabulum (CFH) and cranio-caudal distance of the dorsal acetabular rim (CrCdAR). Radiographs of a total of 264 skeletally mature dogs with similar physical characteristics (German wirehaired pointers (GWP), German shepherd dogs (GSD) and Labrador retrievers (LAB)) presented for routine hip dysplasia screening were recruited for the study. FHA, CFH and CrCdAR were measured and related to dysplasia status. Evaluations of FHA (*p* = 0.011), CFH (*p* < 0.001) and CrCdAR length (*p* = 0.003) measurements revealed significant interactions between breed, sex and FCI scores, so they had to be assessed separately. The results revealed that FHA tends to decrease as the hip dysplasia score worsens. There was no significant relationship between FHA and dysplasia assessment. FHA is breed-specific and is larger in normal and near-normal male (*p* = 0.001, *p* = 0.020) and female (*p* = 0.001, *p* = 0.013) GWP compared to GSD, respectively. FHA is greater in normal male GWP (*p* = 0.011) and GSD (*p* = 0.040) compared to females. There was a significant and strong positive correlation between FHA and CrCdAR in all breeds and sexes. Additionally, FCI scoring had a medium (GWP, GSD) to strong (LAB) negative correlation with CFH.

## 1. Introduction

Canine hip dysplasia (CHD) is a multifactorial developmental disease with strong genetic background encountered in many large dog breeds leading to lameness and degenerative joint disease caused by joint instability and incongruent joint conformation. Early detection of the condition is unreliable [1,2] and causes economic losses especially in competing dogs. Investigation for CHD in skeletally mature dogs is performed by taking radiographs of the hips with extended femora. Screening associations such as the Fédération Cynologique Internationale (FCI) evaluate hip joint morphology, presence or absence of signs of osteoarthritis, joint laxity, and Norberg angle (NA) [3,4,5]. Femoral head coverage, defined as the overlap of the femoral head by the dorsal acetabular roof, has also been evaluated as a parameter to evaluate CHD status [6,7,8] and helps in assessing success rates after pelvic surgeries [9,10]. Coxo-femoral joint incongruence is an important radiographic finding and criterion for the scoring and assignment to FCI dysplasia categories. Joint incongruence may be a consequence of both joint laxity resulting from increased elasticity of the soft tissues of the joint and/or size/shape incompatibility of the femoral head and acetabulum. Femoral head shape has been related to CHD [11] with poor correlation; however, to the best of our knowledge, femoral head size has not been measured and associated with CHD, although this is also expected to affect the NA. Studies on artificial intelligence and machine learning are emerging in the field of medical imaging to aid in hip screening applications [12]. Morphological studies are needed to validate certain programs, define certain patterns and select appropriate candidates (breeds) for training.

The purpose of this study was to find out whether femoral head area (FHA), percentage coverage of the femoral head (CFH), and length of the cranio-caudal dorsal acetabular rim (CrCdAR) were related to CHD investigated in three dog breeds with relatively high prevalence and risk for CHD [13]. The effect of breed and sex on these parameters was also of interest. Further, it needed to be determined whether it made a difference to compare dysplastic dogs (FCI-C/D/E) with non-dysplastic dogs comprising only FCI-A scores or also include those with FCI-B scores assigning them as “non-dysplastic” [2].

## 2. Materials and Methods

Client-owned German wirehaired pointers (GWP), German shepherd dogs (GSD) and Labrador retrievers (LAB) of both sexes presented for routine CHD screening were included in the study. The animals recruited for the study were skeletally mature, ranging in age between 1–4 years, and the mean age was 1.9 years (median 2), 1.8 (median 1) and 1.2 years (median 1) in the GWP, GSD and LAB, respectively. Mean bodyweight was 28.7 ± 2.9 kg in GWP, 29.4 ± 4.3 kg in GSD and 31.8 ± 4.1 kg in LAB.

**Figure 1:**
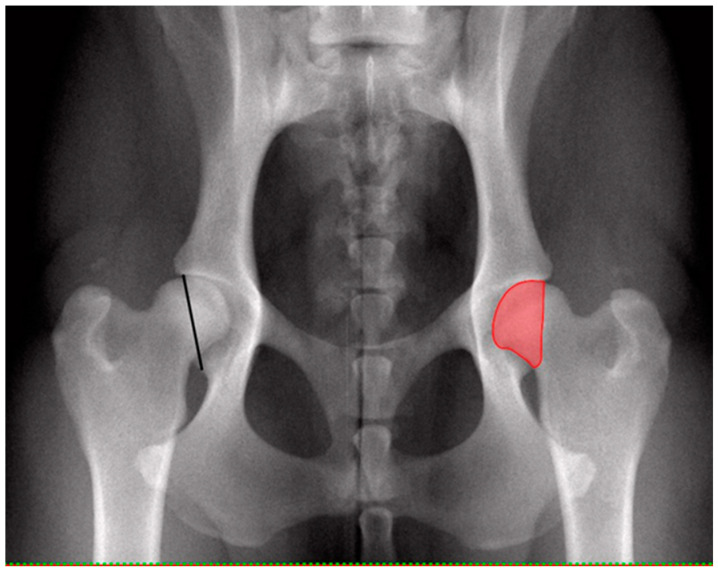
Ventrodorsal pelvic radiograph of normal coxofemoral joints (left) indicating the cranio-caudal distance of the dorsal acetabular rim (CrCdAR), the shortest distance (black line) between the craniolateral edge and the caudolateral edge of the acetabulum and (right) surface coverage of femoral head (CFH, red area).

Radiographs were taken with digital radiography in symmetrical ventrodorsal projection with extended, parallel femora and patellae in the middle of the femoral condyles while dogs were under general anesthesia with muscle relaxation achieved by a routine injectable anesthetic protocol. Hip joints were evaluated for dysplasia based on FCI criteria from A to E [3,4,5] for every hip joint separately by blinded authorized investigators (DSI, BT). DICOM reader software (Version V3.3.6, Horos, for Apple Macintosh) was utilized to determine femoral head area (FHA) and percentage femoral coverage by the acetabulum (CFH). For this purpose, measurements were performed by a trained postgraduate student (MP) blinded to dysplasia scores. The diameter, circumference and FHA were determined by drawing the best fitting circle around the femoral head using a circle template with a line of least thickness. The overlap of FHA and dorsal acetabular roof (surface femoral coverage) was determined by drawing a line along the dorsal acetabular rim connecting to the circle of the femoral head cranially and caudally. Area calculations were performed by the software and recorded in square centimeters. Percentage CFH was calculated manually by dividing FHA superimposed by the acetabulum by the total FHA and multiplied by 100 [6,8,9]. Each individual hip score was related to FHA and CFH. Comparisons were made between dysplasia-free and dysplastic hips. Dysplasia-free was defined as hips having a score A, while dysplastic was defined as having mild, moderate or severe CHD (score C–E). Additionally, the shortest distance between the cranio-lateral edge and the caudo-lateral edge of the acetabulum was measured with the distance tool of the DICOM reader software and related to the corresponding FHA (Figure 1).

The data of the present study were analyzed using the IBM SPSS Statistics 23.0 program. Mean and standard deviation values were given for the quantitative (continuous) variables used in the research. The normality of variables was evaluated using the Komogorov–Smirnov test. A linear mixed model was used to evaluate the relationship between femoral head area (FHA), coverage of the femoral head (CFH) and cranio-caudal acetabular rim (CrCdAR) length with dysplasia status (FCI). For this purpose, the right and left measurements of the assessed bones were included in the model as a random effect subject. The model also included the influence of three different breeds and two sexes. FCI score was modeled as A, B, C, D and E levels. Breed, sex and FCI score were considered as fixed factors, and specific relationship effects were included in the model as well as main effects of the factors. In the first stage, the specific relationship effects were evaluated, and if this effect was found to be significant, the main effects were not evaluated. Significant differences were determined by the Sidak post hoc test with Bonferroni correction. In addition, the relationship of FHA, CFH, and CrCdAR length with FCI score was evaluated separately for each breed and sex with Spearman’s rank correlation analysis. The statistical significance level was determined as α = 0.05. *p*-values below this value were considered statistically significant (*p* < 0.05), and *p* values above this value were considered statistically insignificant (*p* > 0.05).

## 3. Results

A cohort of 264 dogs including GWP, GSD and LAB of both sexes were evaluated. Breed and sex distribution of the dogs are shown in Table 1.

Hip scores with breed and sex distribution are shown in Table 2. The cohort revealed a prevalence of CHD in GWP, GSD and LAB of 11.6%, 35.8% and 44.3%, respectively.

### 3.1. Femoral Head Area (FHA)

As a result of the evaluations for FHA measurements, it was observed that there was a specific relationship between breed, sex and FCI score (*p* = 0.011). As the relationship was significant, comparisons for each breed and sex with FCI levels in terms of FHA had to be evaluated separately. Therefore, in Table 3, FHA averages are given separately for each breed, sex and FCI level. As can be seen from the numbers listed in Table 2, there were no GWP breed males with FCI level E and no GWP females with FCI level D or E. Male and female GSD and LAB had all levels of FCI scoring. There were no dogs for the rows indicated with (--) in Table 3.

In Table 4, FCI levels (A to E) are compared in terms of FHA measurements, separately for each breed and sex, and the *p*-values for the differences between the levels are included. As a result of the evaluations, there was no significant difference between FCI levels among male and female dogs. In male GSD, the mean FHA in those with FCI levels A, B, C and D was found to be significantly smaller than in those with E level. No other significant difference was found. No significant difference was found between FCI levels in female GSD. There was no significant difference between FCI levels in both male and female LAB.

According to the results obtained, while the mean FHA in those with FCI level E was found to be significantly larger than in those with other FCI levels only in GSD and male sex, the results of FCI comparisons in all other breeds and sexes were not significant. In this case, it was decided that FCI level E could be differentiated from other levels by looking at FHA measurements in male dogs of the GSD breed. However, in other conditions, the FHA level did not have a distinctive role in terms of disease severity.

Table 5 shows the significance of the interracial differences for each sex and FCI level.

As a result of the evaluations, it could be said that there was a significant difference between GWP and GSD and between GWP and LAB in males with an FCI result of A, and the mean FHA was significantly larger in the GWP breed. There was a significant difference between all three breeds in males with FCI level B, and the highest mean was found in GWP, followed by GSD and the lowest in LAB. No interbreed differences were found in male dogs with FCI level C. There was a significant difference between GWP and GSD and between GWP and LAB in male dogs with FCI level D, but there was no significant difference between GSD and LAB. FHA values for male GSD were significantly larger in FCI-E dogs compared to related LAB. There was only a significant difference between GWP and GSD and between GWP and LAB in female dogs with FCI level A and FCI level B. In females with FCI level C, there was a significant difference only between GWP and LAB, and the mean FHA was significantly larger in the GWP breed.

Table 6 shows significant differences between the sexes separately for each breed and FCI level. As a result of the evaluations, the mean FHA of males was significantly larger in GWP and those with FCI level A, but there was no significant difference between sexes in this breed among those with FCI levels B and C. The mean FHA of males was significantly larger in GSD and those with FCI levels A and E, but there was no significant difference between sexes in those with FCI levels B, C and D in this breed. There was no significant difference between the sexes in the LAB breed with FCI levels A–E.

The findings in Table 4, Table 5 and Table 6 were the results obtained for five different FCI levels (A to E). In particular, Table 4 lists the results of the comparison of FCI levels in each breed and sex in terms of mean FHA and shows the role of FHA in the diagnosis of the disease. Table 5 and Table 6 explain breed and sex differences in terms of FHA.

### 3.2. Coverage of Femoral Head Area (CFH)

As a result of the evaluations for CFH measurements, it was observed that there was a specific relationship between breed, sex and FCI score (*p* < 0.001). As the relationship was significant, comparisons for each breed and sex with FCI levels in terms of CFH had to be evaluated separately. Therefore, in Table 7, CFH averages are given separately for each breed, sex and FCI level. As can be seen from the numbers in Table 2, there were no GWP breed males with FCI level E and no GWP females with FCI level D or E. Male and female GSD and LAB had all levels of FCI scoring. There were no dogs for the rows indicated with (--) in Table 7. When a cut-off value of 50% was selected to distinguish dysplastic from non-dysplastic dogs, among the FCI-A scored dogs, 3% of GWP, 5% of GSD and none of LAB were misclassified.

In Table 8, FCI levels (A to E) are compared in terms of CFH measurements, separately for each breed and sex, and the *p*-values for the differences between the levels are included. As a result of the evaluations, a significant difference was found between FCI levels A and C and between A and B in males and females of GWP race, and there were significant differences between A and D and B and D in males. It was seen that the mean was higher in A, followed by B, C and D, respectively. In male GSD, the mean CFH of those with FCI levels A, B, C and D was found to be significantly higher than the mean CFH of those with E level. There was also a significant difference between A and D. Apart from this, no significant difference was found. In female GSD, the mean CFH of those with FCI levels A, B, C and D was found to be significantly lower than the mean CFH of those with E level. There was a significant difference between A and C, A and D, and B and D. No other significant difference was found. All FCI levels were found to be significantly different from each other in male LAB; in females, only the difference between B and C was not significant, whereas the other differences were found to be significant.

Comparisons of sex in terms of mean coverage of femoral head (CFH) were made for each breed and Fédération Cynologique Internationale (FCI) level. There was a significant difference between GWP and LAB (*p* = 0.001) and between GSD and LAB (*p* = 0.006) in males with an FCI score of A. There was a significant difference between GWP and LAB (*p* < 0.001) and between GSD and LAB (*p* = 0.001) in females with an FCI score of A. The differences between GWP and GSD (*p* = 0.012) and GSD and LAB (*p* < 0.001) were significant in males with an FCI score of D. It can be said that there was a significant difference between GSD and LAB (*p* = 0.001) in male dogs with FCI level E, and the mean CFH was significantly higher in the GSD breed. Remaining differences were statistically insignificant. The mean CFH of males was significantly higher in only GSD with FCI score of E compared to females.

### 3.3. Cranio-Caudal Acetabular Rim (CrCdAR) Length

As a result of the evaluations for CrCdAR measurements, it was observed that there was a specific relationship between breed, sex and FCI score (*p* = 0.003). As the relationship was significant, comparisons for each breed and sex with FCI levels in terms of CrCdAR length needed to be evaluated separately. Therefore, in Table 9, CrCdAR length averages are given for each breed, sex and FCI score. As can be seen from the numbers shown in Table 2, there were no GWP breed males with an FCI score of E, and no GWP females with an FCI score of D or E. Male and female GSD and LAB had all levels of FCI scoring. There were no dogs for the rows indicated with (--) in Table 9.

In terms of CrCdAR measurements, FCI levels (A to E) were compared for each breed and sex separately. As a result of the evaluations, in males (*p* = 0.049) and females (*p* = 0.050) of GWP, CrCdAR length only in those with FCI level A was found to be significantly larger than in those with FCI-B. The mean CrCdAR length in GSD females with FCI levels A, B, C (*p* = 0.001 for each) and D (*p* = 0.020) was significantly larger than in those with FCI level E. In females of the LAB breed, the mean CrCdAR length only in those with FCI level E was found to be significantly larger than in those with FCI-A (*p* = 0.048). Apart from these, no significant difference was found between FCI levels.

CrCdAR measurements of FCI-A GWP were found to be significantly larger than those of LAB in males (*p* = 0.006) and females (*p* < 0.001). In FCI-B dogs, values of GWP (*p* < 0.001) and GSD (*p* = 0.003) were significantly larger than those of LAB. In FCI-D dogs, CrCdAR lengths in male GWP were significantly larger compared to those of LAB (*p* = 0.004). CrCdAR lengths in FCI-E scored male GSD were significantly larger compared to those of LAB (*p* = 0.006).

Comparison of mean CrCdAR values in male and female dogs revealed that values of FCI-A males in all three breeds (GWP: *p* = 0.002; GSD: *p* = 0.005; LAB: *p* < 0.001) were significantly greater than those of females. Additionally, FCI-B male GWP values were significantly greater than those of females.

Table 10 shows the correlations between FHA, CFH and CrCdAR measurements and FCI evaluations by breed and sex. Accordingly, except for GWP and LAB males that showed a weak negative correlation (the smaller FHA, the worse the FCI score), there was no significant correlation between FCI scores and FHA. The negative correlation (the smaller CFH, the worse the FCI score) between FCI scoring and CFH was moderate in GWP and GSD and high in LAB in both sexes. Correlations between FCI scoring and CrCdAR lengths were inconsistent in males and females of all breeds. Similarly, there was no correlation between FHA and CFH. However, the FHA and CrCdAR length showed a high positive correlation in all breeds and sexes, apart from female LAB, who showed a moderate correlation.

One of our objectives was to investigate if it made a difference to compare dysplastic dogs (FCI-C/D/E) with non-dysplastic dogs comprising only FCI-A scores or to also include dogs with FCI-B scores by assigning them as "non-dysplastic". However, in all parameters, statistical tests revealed an interaction between breed, sex and FCI scores that rendered merging FCI-A dogs and FCI-B dogs into one healthy group impossible.

## 4. Discussion

CHD is defined as an inherited incongruity of the coxofemoral joint caused by excessive laxity of joint stabilizing soft tissues ending in osteoarthrosis and pain due to abnormal pressure and wear with subsequent new bone formation in certain regions of the joint. In addition to joint laxity, another reason for joint incongruity could also be a disproportion of the bony components relative to each other [3,4,14,15]. Thus, one of the objectives of the present study was to find out if the femoral head size showed any differences among FCI dysplasia status groups and to see if there was any relation of FHA to the CrCdAR length as a morphometric parameter of the acetabular size. Further, the influence of breed and sex on FHA, CFH and CrCdAR was investigated.

The prevalence of CHD in our dog population was closer to the upper limit compared to that of other studies [16,17], although studies showed that some improvement has been achieved over the years [18]. The discrepancy can be attributed to the difficulty in recruiting a typical sample of the total population and may also be due to differences in the prevalence of CHD in local dog populations. GWP dogs that had generally lower dysplasia prevalence and better FCI scores than GSD and LAB [13] showed a significantly larger FHA compared to t FCI-A and FCI-B scored dogs in the other breeds. Generally, there was a tendency of decreasing FHA values towards worse FCI scoring in both sexes. Exceptions were male FCI-D GWP dogs and FCI-E GSD dogs of both sexes and female LAB. This could be explained by the low case number and a high standard deviation rate. FHA was not found useful in distinguishing FCI levels, as only male GSD with FCI-E hips were significantly different from all the others. So, the FHA level did not have a distinctive role in terms of disease severity. Wigger et al. [11] investigated broomstick-shaped femoral heads, which were poorly or even not demarcated from the femoral neck and appeared relatively smaller than in other hips. However, no measurements were made to prove this in that study, and broomstick-shaped femoral heads did not appear to be associated with the occurrence of CHD.

Nevertheless, in our study there were some significant breed differences that were observed between GWP, GSD and LAB, although these breeds are similar in bodyweight and size. The FHA of male and female GWP in the normal and near-normal (FCI-A and B) categories were significantly larger than those of GSD and LAB. Differences between GSD and LAB were less significant.

Sex differences were only obvious in FCI-A dogs of GWP and GSD. While some studies observed no sex differences, others detected a higher prevalence of CHD in females with a variability by breed [19]. In the present study, male and female GWP and LAB had nearly the same CHD prevalence, but female GSD were twice as often affected by CHD compared to male GSD. This may suggest that females with a smaller FHA may be more prone to CHD compared to males. Studies comparing human and canine patients found that affected individuals were 80% female in humans, while in dogs, no sex predilection was proven [20,21]. Sex was also not a significant risk factor for DJD associated with CHD [22].

Investigations of CFH measurements revealed significant specific relationships in terms of breed and FCI evaluations. Several screening systems [3] and evaluation criteria [6,7,8,23] have been developed and employed for the selection of dogs for breeding, mainly taking signs of osteoarthritis into account. The coverage area of the femoral head by the dorsal aspect of the acetabulum is another parameter to assess hip joints for CHD. Besides the NA, percentage CFH is a measurable parameter with a suggested cutoff value of approximately ≥50% considered to be normal, while less is considered to indicate joint incongruity and dysplasia [7,24]. However, some studies found that there are breed specific differences [7,8] that could lead to a too strict evaluation, eliminating dogs from the breeding pool. They recommend judging dogs individually as a breed instead of using universal criteria [8,25,26]. Tomlinson and Johnson [8] suggested a more accurate value of 42.2% for LAB and 44.8% for GSD, while Mostafa et al. [27] suggested a cut-off value of <53% for the dorsal acetabular femoral head coverage area index for LAB. In our study, a limit of 45% revealed an improved evaluation of two GSD dogs classified as FCI-A (results not shown here). This could be due to a retracted concave dorsal acetabular rim, which is sometimes observed. This might cause a smaller coverage area while other features of the joint are within normal limits. To our knowledge there are no published breed-specific data on CFH in GWP. So, in the present study, we preferred to stay with 50% as a cut-off value, a middle ground. Herewith, the FCI-A group showed 3% false positive cases in GWP, 5% in GSD, and none in LAB. CFH measurements revealed a significant relationship between breed, sex and FCI score. In male and female GWP, the CFH values of FCI-A and B were not significantly different, indicating that radiographic features were similar. However, FCI-A and B were significantly different from those of FCI-C and D in both sexes (there were no FCI-E GWP). This would mean that at least a distinction between dysplastic and non-dysplastic is reliable for GWP. In GSD, the gray-zone between dysplastic and non-dysplastic seems to be less clear, because only FCI-E hips are significantly different from those with other scores, and the remaining grades were inconsistent, with a slightly better result in females. In LAB, however, there was a clear and highly significant distinction between all FCI scores in both sexes, which makes this parameter quite valuable in terms of CHD assessment. Thus, with this feature, the LAB breed may also be considered suitable for training with machine learning.

The CrCdAR was meant to be a parameter of the acetabular size, the counterpart of the femoral head. The study needed to determine if there was a relationship between the CrCdAR length and FHA and/or CFH. It was observed that there was a specific relationship between breed, sex and FCI score. However, CrCdAR length could not be validated as a distinctive variable for CHD in terms of FCI grading, and significance levels according to breed and sex were inconsistent. FCI-A dogs had significantly greater CrCdAR lengths compared to FCI-B, C and D dogs, while differences between these FCI groups were insignificant. FCI-E dogs, however, also showed significantly greater CrCdAR lengths. Breed differences were only significant between LAB and GWP or GSD, while values for males were larger than those in females. Fealey et al. [28] investigated canine pelvic morphology and found significant differences between principal components in males and females, validating sexual dimorphism of the pelvis. Male dogs had significantly larger pelvises than bitches. They observed that dogs with larger pelvises tended to have smaller NAs and in turn were more likely to have CHD. It is well-known that large-breed dogs show a higher prevalence of CHD compared to smaller breeds [29], which may be attributable to larger pelvises with larger femoral heads and smaller NAs. There is also a strong positive correlation of relative body length with a higher prevalence of CHD in certain dog breeds [30]. In a recent study, investigators [31] developed and validated a new index (Hip Congruency Index; HCI) to assess hip joint congruity and to incorporate this parameter into an artificial intelligence algorithm. The CFH was related to the acetabular area, and the resulting index decreased gradually with statistically significant differences between all FCI categories.

In the present study, hip joints were evaluated for dysplasia based on FCI criteria from A to E [3] for every hip joint separately, as hip dysplasia expression may differ between hips [19] and, therefore, the relationship between FHA/CFH and CrCdAR length. To find the association between these parameters, related correlation coefficients were calculated. There was a strong positive correlation between FHA and CrCdAR length in all dog breeds and sexes. Thus, it can be expected that with larger femoral heads, the acetabulum becomes larger as well. However, FHA and FCI scores did not manifest a significant correlation in any group. The theory that aside from excessive laxity of joint-related soft tissue structures, reciprocal disproportional bony components could also contribute to incongruent joint conformation was not supported by the results of the present study.

## 5. Conclusions

FHA and CFH values are breed-specific and are both greater in normal males compared to females in GWP and GSD. FHA has a strong positive correlation with CrCdAR length, suggesting that bony components of the hip joints maintain a certain proportion reciprocally. FCI hip score groups did not reveal a consistently significant difference concerning FHA and CFH. However, certain tendencies may encourage further studies with a larger and more balanced caseload.

## Figures and Tables

**Table 1:** Breed and sex distribution of dogs.

	GWP (n)	GSD (n)	LAB (n)	Total
Male	43	34	51	128
Female	43	47	46	136
Total	86	81	97	264

GWP, German wirehaired pointers; GSD, German shepherd dogs; LAB, Labrador retrievers.

**Table 2:** Distribution of Fédération Cynologique Internationale (FCI) dysplasia status of hip joints with respect to breed and sex in German wirehaired pointers (GWP), German shepherd dogs (GSD) and Labrador retrievers (LAB).

FCI Score	Male				Female			
	GWP	GSD	LAB	Total	GWP	GSD	LAB	Total
A	39	21	24	84	51	18	27	96
B	37	32	32	101	25	33	25	83
C	5	5	24	34	10	20	27	57
D	5	6	16	27	0	12	9	21
E	0	4	6	10	0	11	4	15
Total	86	68	102	256	86	94	92	272

FCI, Fédération Cynologique Internationale; GWP, German wirehaired pointers; GSD, German shepherd dogs; LAB, Labrador retrievers.

**Table 3:** Mean values (cm^2^) of femoral head area (FHA) sorted by breed, sex and Fédération Cynologique Internationale (FCI) results.

Breed	FCI	Male			Female		
		n	Mean	SD	n	Mean	SD
GWP	A	39	4.416	0.749	51	4.015	0.697
	B	37	4.034	0.914	25	3.877	0.693
	C	5	3.735	0.645	10	3.662	0.836
	D	5	4.292	0.700	0	--	--
	E	0	--	--	0	--	--
GSD	A	21	3.701	0.676	18	3.208	0.429
	B	32	3.544	0.793	33	3.313	0.722
	C	5	3.547	0.672	20	3.235	0.569
	D	6	3.116	0.737	12	3.099	0.469
	E	4	5.295	1.841	11	3.773	1.027
LAB	A	24	3.397	0.388	27	3.098	0.526
	B	32	3.046	0.641	25	3.341	1.033
	C	24	3.055	0.505	27	2.965	0.674
	D	16	2.886	0.488	9	3.002	0.870
	E	6	2.913	0.287	4	3.647	1.374

FCI, Fédération Cynologique Internationale; GWP, German wirehaired pointers; GSD, German shepherd dogs; LAB, Labrador retrievers; SD, standard deviation.

**Table 4:** Comparison of Fédération Cynologique Internationale (FCI) levels in terms of mean femoral head area (FHA) for each breed and sex.

Breed	Sex	FCI	FCI			
			B	C	D	E
			*p*-Values			
GWP	Male	A	0.122	0.254	0.999	
		B		0.947	0.973	
		C			0.781	
		D				
	Female	A	0.820	0.405		
		B		0.813		
		C				
		D				
GSD	Male	A	0.997	1.000	0.570	0.001
		B		1.000	0.868	0.000
		C			0.98	0.003
		D				0.000
	Female	A	1.000	1.000	1.000	0.346
		B		1.000	0.991	0.506
		C			1.000	0.386
		D				0.230
LAB	Male	A	0.529	0.657	0.261	0.785
		B		1.000	0.999	1.000
		C			1.000	1.000
		D				1.000
	Female	A	0.924	0.999	1.000	0.820
		B		0.470	0.925	0.997
		C			1.000	0.561
		D				0.774

FCI, Fédération Cynologique Internationale; GWP, German wirehaired pointers; GSD, German shepherd dogs; LAB, Labrador retrievers.

**Table 5:** Comparison of breeds in terms of mean femoral head area (FHA) for each sex and Fédération Cynologique Internationale (FCI) level.

Sex	FCI	Breed	Breed	
			GSD	LAB
			*p*	*p*
Male	A	GWP	0.001	0.000
		GSD		0.409
	B	GWP	0.016	0.000
		GSD		0.018
	C	GWP	0.968	0.159
		GSD		0.422
	D	GWP	0.022	0.001
		GSD		0.885
	E	GWP		
		GSD		0.000
Female	A	GWP	0.000	0.000
		GSD		0.944
	B	GWP	0.010	0.027
		GSD		0.999
	C	GWP	0.335	0.028
		GSD		0.500
	D	GWP		
		GSD		0.761
	E	GWP		
		GSD		0.764

FCI, Fédération Cynologique Internationale; GWP, German wirehaired pointers; GSD, German shepherd dogs; LAB, Labrador retrievers.

**Table 6:** Comparison of sexes in terms of mean femoral head area (FHA) for each breed and Fédération Cynologique Internationale (FCI) level.

Breed	FCI	Sex	Sex
			Female
			*p*
GWP	A	Male	0.009
	B	Male	0.403
	C	Male	0.854
	D	Male	
	E	Male	
GSD	A	Male	0.034
	B	Male	0.199
	C	Male	0.388
	D	Male	0.963
	E	Male	0.000
LAB	A	Male	0.141
	B	Male	0.127
	C	Male	0.657
	D	Male	0.709
	E	Male	0.116

FCI, Fédération Cynologique Internationale; GWP, German wirehaired pointers; GSD, German shepherd dogs; LAB, Labrador retrievers.

**Table 7:** Mean values (%) for coverage of femoral head (CFH) sorted by breed, sex and Fédération Cynologique Internationale (FCI) results.

Breed	FCI	Male			Female		
		n	Mean	SD	n	Mean	SD
GWP	A	39	60.675	5.686	51	60.074	5.509
	B	37	57.117	6.133	25	56.811	4.775
	C	5	47.521	7.085	10	48.702	6.309
	D	5	40.117	8.805	0		
	E	0	--		0		
GSD	A	21	61.046	5.377	18	59.875	5.442
	B	32	57.945	6.459	33	54.906	5.992
	C	5	54.656	8.309	20	52.693	8.777
	D	6	52.102	8.877	12	47.019	10.381
	E	4	37.445	6.059	11	28.515	16.268
LAB	A	24	67.325	5.826	27	67.632	8.311
	B	32	56.080	6.791	25	55.587	4.153
	C	24	50.413	6.360	27	51.920	5.671
	D	16	39.162	7.376	9	43.248	8.660
	E	6	22.419	8.468	4	22.520	7.019

FCI, Fédération Cynologique Internationale; GWP, German wirehaired pointers; GSD, German shepherd dogs; LAB, Labrador retrievers; SD, standard deviation.

**Table 8:** Comparison of Fédération Cynologique Internationale (FCI) levels in terms of mean coverage of femoral head (CFH) for each breed and sex.

Breed	Sex	FCI	FCI			
			B	C	D	E
			*p*-Values			
GWP	Male	A	0.132	0.000	0.000	
		B		0.019	0.000	
		C			0.427	
		D				
	Female	A	0.143	0.000		
		B		0.005		
		C				
		D				
GSD	Male	A	0.672	0.461	0.046	0.000
		B		0.977	0.428	0.000
		C			1.000	0.002
		D				0.009
	Female	A	0.123	0.012	0.000	0.000
		B		0.945	0.006	0.000
		C			0.207	0.000
		D				0.000
LAB	Male	A	0.000	0.000	0.000	0.000
		B		0.021	0.000	0.000
		C			0.000	0.000
		D				0.000
	Female	A	0.000	0.000	0.000	0.000
		B		0.419	0.000	0.000
		C			0.010	0.000
		D				0.000

FCI, Fédération Cynologique Internationale; GWP, German wirehaired pointers; GSD, German shepherd dogs; LAB, Labrador retrievers.

**Table 9:** Mean values (cm) of cranio-caudal acetabular rim (CrCdAR) lengths sorted by breed, sex and Fédération Cynologique Internationale (FCI) results.

Breed	FCI	Male			Female		
		n	Mean	SD	n	Mean	SD
GWP	A	39	3.023	0.285	51	2.842	0.252
	B	37	2.859	0.309	25	2.688	0.186
	C	5	2.772	0.106	10	2.681	0.265
	D	5	3.063	0.159	0	--	--
	E	0	--	--	0	--	--
GSD	A	21	2.917	0.313	18	2.671	0.165
	B	32	2.832	0.316	33	2.716	0.237
	C	5	2.757	0.167	20	2.696	0.190
	D	6	2.765	0.329	12	2.735	0.203
	E	4	3.183	0.549	11	3.085	0.322
LAB	A	24	2.805	0.137	27	2.527	0.250
	B	32	2.609	0.335	25	2.675	0.366
	C	24	2.700	0.226	27	2.673	0.198
	D	16	2.615	0.292	9	2.753	0.321
	E	6	2.699	0.250	4	2.935	0.473
Total	A	84	2.934	0.273	96	2.721	0.273
	B	101	2.771	0.335	83	2.695	0.268
	C	34	2.719	0.203	57	2.683	0.204
	D	27	2.731	0.322	21	2.743	0.253
	E	10	2.892	0.445	15	3.045	0.356

FCI. Fédération Cynologique Internationale; GWP. German wirehaired pointers; GSD. German shepherd dogs; LAB. Labrador retrievers; SD. standard deviation.

**Table 10:** Correlations between measurements made separately for each breed and sex and the Fédération Cynologique Internationale (FCI) dysplasia status.

Breed	Sex		FHA			CFH				CrCdAR	
			*N*	*r*	*P*	*N*	*r*	*P*	*N*	*r*	*P*
GWP	Male	FCI	−0.271	0.012	86	−0.512	0.000	86	−0.263	0.014	86
		FHA				0.113	0.301	86	0.840	0.000	86
		CFH							0.122	0.263	86
	Female	FCI	−0.177	0.104	86	−0.468	0.000	86	−0.320	0.003	86
		FHA				0.010	0.928	86	0.740	0.000	86
		CFH						86	−0.029	0.792	86
GSD	Male	FCI	−0.034	0.782	68	−0.473	0.000	68	−0.080	0.518	68
		FHA				−0.134	0.276	68	0.828	0.000	68
		CFH						68	−0.039	0.752	68
	Female	FCI	0.037	0.721	94	−0.560	0.000	94	0.250	0.015	94
		FHA				−0.110	0.293	94	0.748	0.000	94
		CFH						94	−0.236	0.022	94
LAB	Male	FCI	−0.388	0.000	102	−0.843	0.000	102	−0.137	0.170	102
		FHA				0.277	0.005	102	0.725	0.000	102
		CFH						102	0.144	0.148	102
	Female	FCI	−0.184	0.079	92	−0.784	0.000	92	0.334	0.001	92
		FHA				0.001	0.990	92	0.555	0.000	92
		CFH						92	−0.185	0.077	92

FCI, Fédération Cynologique Internationale; GWP, German wirehaired pointers; GSD, German shepherd dogs; LAB, Labrador retrievers; FHA, Femoral head area; CFH, Femoral coverage by the acetabulum; CrCdAR, cranio-caudal acetabular rim length.

## Data Availability

Not applicable.
